# Green Earth pigments aqueous dispersions: NMR relaxation rates dataset

**DOI:** 10.1016/j.dib.2020.106270

**Published:** 2020-09-03

**Authors:** Agathe Fanost, Maguy Jaber, Laurence de Viguerie, Jean-Pierre Korb, Pierre E. Levitz, Laurent J. Michot, Guillaume Mériguet, Anne-Laure Rollet

**Affiliations:** a Laboratoire PHysico-chimie des Electrolytes et Nanosystèmes InterfaciauX, PHENIX, Sorbonne Université, CNRS, F-75005 Paris, France; b Laboratoire d'Archéologie Moléculaire et Structurale, LAMS, Sorbonne Université, CNRS, F-75005 Paris, France

**Keywords:** Celadonite, Glauconite, Phyllosilicate, Fast Field Cycling NMR relaxometry, Water surface interaction

## Abstract

The data presented here are related to the research paper entitled “Green Earth pigments dispersions: water dynamics at the interfaces”. The nuclear magnetic resonance (NMR) relaxometry data are provided for various aqueous Green Earth (GE) pigments dispersions with volume fraction spanning approximately from 0.1 to 0.5. For two of them (Cyprus GE and Bohemian GE), the NMR relaxation profiles from 10 kHz to 30 MHz (^1^H frequency) is given for several temperatures spanning from 293 to 318K. In addition, the X-ray diffraction pattern is provided for France GE (Kremer pigments) for the identification of the main mineral component. The nitrogen gas isotherms are provided for Cyprus GE and Bohemian GE.

## Specifications Table

**Table:** 

Subject	Physics – Surfaces and interfaces
Specific subject area	Solvent Diffusion and interaction with inorganic surfaces probed by NMR relaxometry
Type of data	Table Graph Image
How data were acquired	Nuclear Magnetic Resonance (NMR), X-ray diffraction (XRD), Gas isotherm NMR relaxometer Stelar FFC spinmaster, NMR relaxometer minispec Bruker 20 MHz, X-ray Diffractometer Bruker D8, Micromeritics ASAP 2020 instrument Software: AcqNMR (Stelar relaxometer) and Minispec
Data format	NMR profile: .sdf (text); R1 at 20MHz: excel file .xlsx; gas absorption: excel file .xlsx; X-ray diffractogram: bruker file .uxd Raw and Analyzed
Parameters for data collection	Data were collected in laboratory-controlled conditions for temperature (293–318K), water content (35–80 water weight%), pressure (1 atm).
Description of data collection	The green earth pigments dispersions were characterized by nuclear magnetic resonance relaxometry at variable field. Mineral composition was determined using X-ray diffraction on powder. Specific surface areas were determined using N_2_ absorption.
Data source location	Institution: Sorbonne Université / CNRS City/Town/Region: Paris Country: France
Data accessibility	Repository name: Mendeley Data Data identification number: Mendeley Data, V1, doi: 10.17632/r9kht3pbn9.1 Direct URL to data: http://dx.doi.org/10.17632/r9kht3pbn9.1
Related research article	Green Earth pigments dispersions: water dynamics at the interfaces A. Fanost, M. Jaber, L. de Viguerie, J.-P. Korb, P. E. Levitz, L. J. Michot, G. Mériguet, A.-L. Rollet, Journal of Colloid and Interface Science. 2021, 581, 644–655 (http://dx.doi.org/10.1016/j.jcis.2020.07.085)

## Value of the Data

• NMR relaxometry data provide a comprehensive insight into the multiscale dynamics of water in concentrated natural phyllosilicate dispersions.
• Data could benefit researches on clay science and on natural pigments used in the field of cultural heritage as they give insight into the wettability of phyllosilicates surfaces (edge and basal) for water.
• The data allow to understand the interaction of water with the different kinds of phyllosilicate surfaces (basal and edge).
• The data allow to characterize the quality of natural phyllosilicate dispersions.

## 1 Data Description

**Fig. 1:**
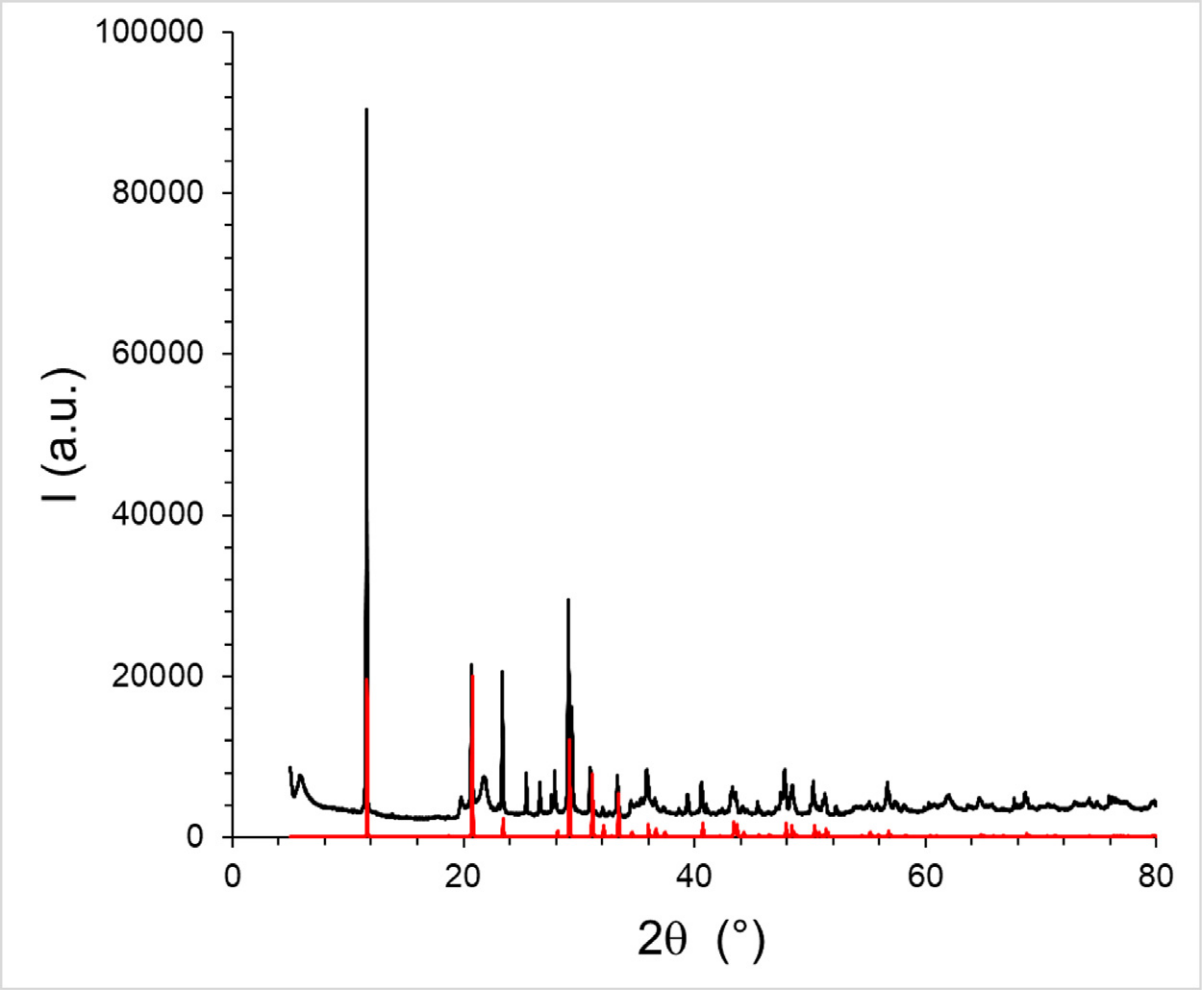
XRD pattern of France GE powder (black) and calculated XRD pattern of CaSO_4_,2H_2_O from the CIF file [2].

**Fig. 2:**
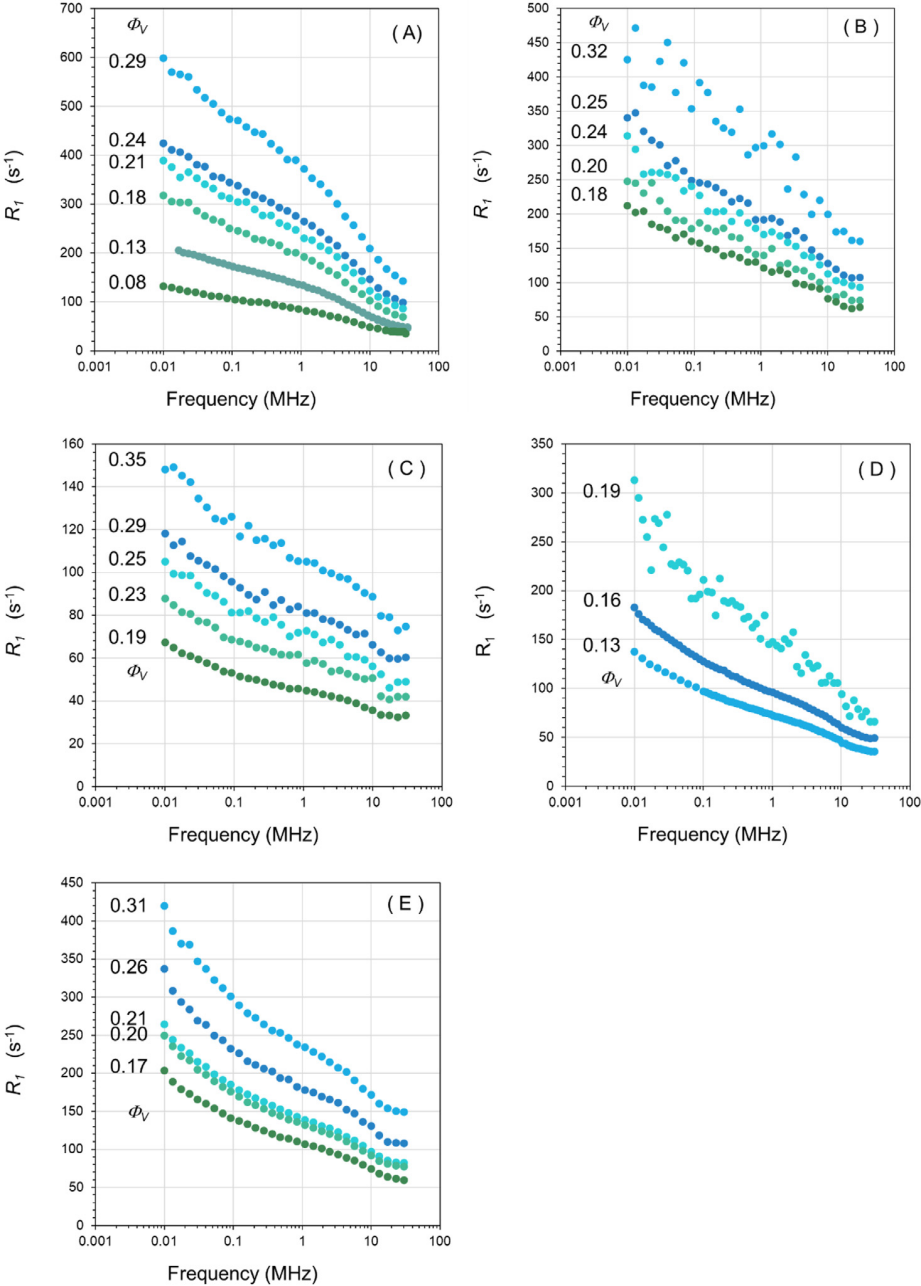
^1^H relaxation rate R_1_ dispersion profile of water in several GE dispersions for several pigment volume fraction: (A) Cyprus GE, (B) Cyprus GBE, (C) Russia GE, (D) France GE and (E) Sennelier GE.
- ^1^H relaxation rate *R_1_*=1/*T_1_* at 0.47 T of water in six GE dispersions as a function of pigment volume fraction (Fig. 3): Bohemian GE, Cyprus GE, Cyprus GBE, Russia GE, France GE and Sennelier GE.

The article presents physico-chemical data about aqueous concentrated dispersions of various natural green earth (GE) pigments. These GE pigments are: Cyprus GE (Kremer Pigmente K17400), Cyprus green blue earth (GBE) (Kremer Pigmente K17410), Russia GE (Kremer Pigmente K11110), Bohemian GE (Kremer Pigmente K40810), Sennelier GE (Sennelier 213) and France GE (Kremer Pigmente K40810). The pigments are mainly composed of phyllosilicate mixtures (celadonite, glauconite, montmorillonite). Their main mineral composition is listed in Table 1 of JCIS article according to Fanost et al. [1]. As France GE was not studied in Fanost et al. paper, X-ray diffraction pattern of France GE is presented in Fig. 1 to identify the main mineral component. The average chemical composition of each mineral is given in Table 1. The data presented in this paper consist of:

- ^1^H NMR relaxometry profiles of five GE (Fig. 2): Cyprus GE, Cyprus green blue earth (GBE), Russia GE, France GE and Sennelier GE.

**Fig. 3:**
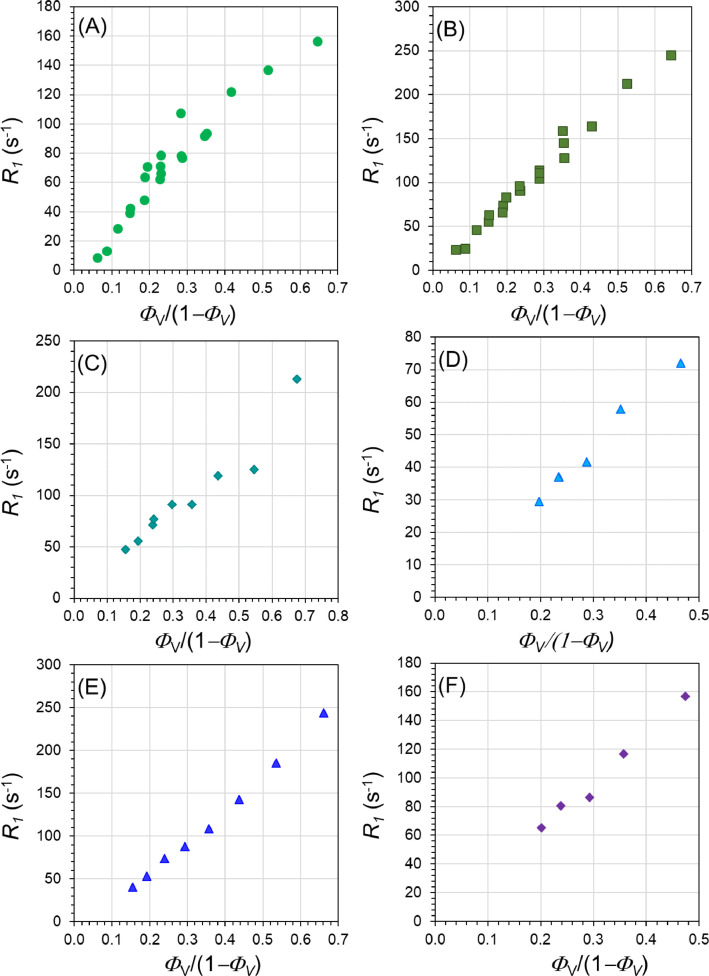
Variation of R_1_ as a function of Φ_V_ at 20 MHz for different GE dispersions: (A) Bohemian GE, (B) Cyprus GE, (C) Cyprus GBE, (D) Russia GE, (E) France GE and (F) Sennelier GE.
- N_2_ gas isotherm of Bohemian GE and Cyprus GE (Fig. 4).

**Fig. 4:**
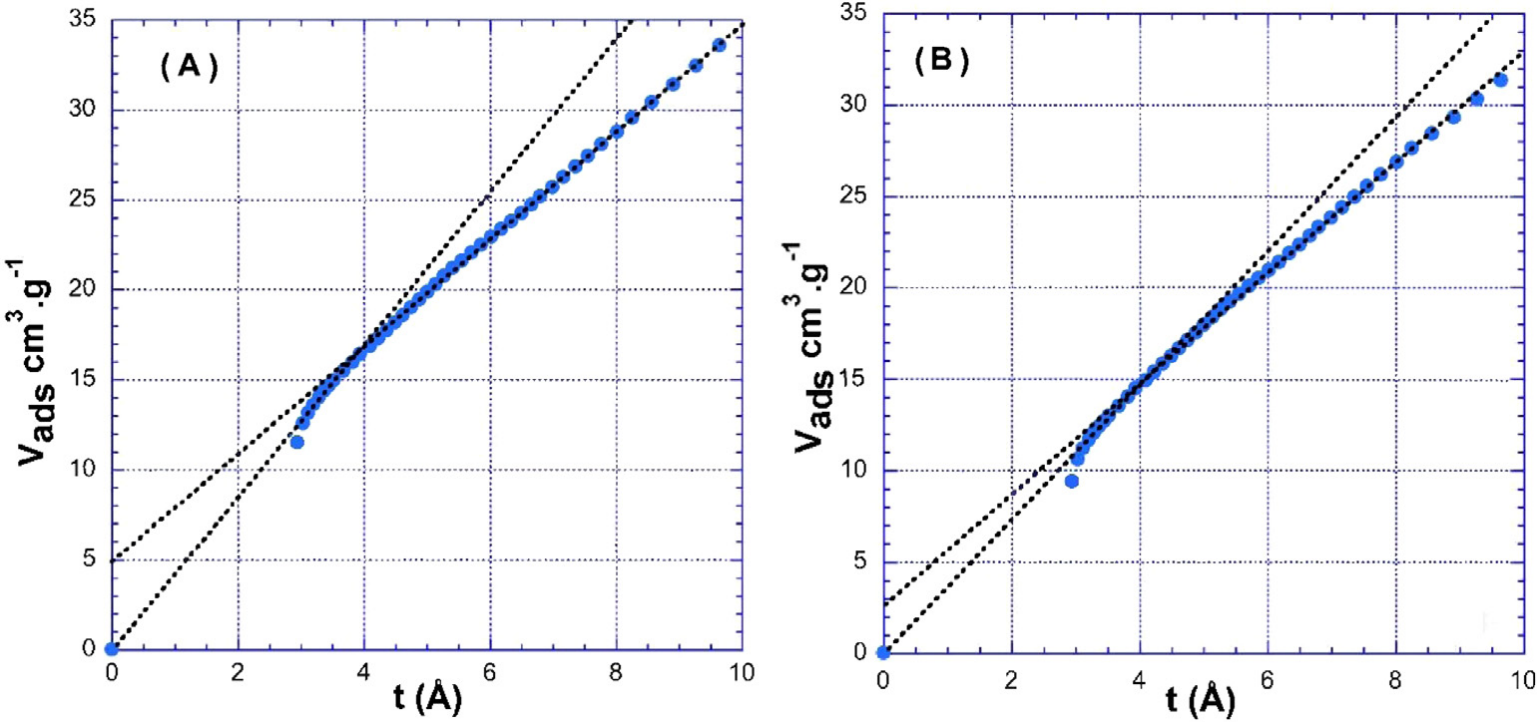
t-plot curves obtained for Bohemian GE (A) and Cyprus GE (B).

**Table 1:** chemical theoretical composition of the different minerals

Mineral	Chemical composition
Celadonite	K (Fe^3+^,Al^3+^) (Mg^2+^, Fe^2+^)☐ [Si_4_O_10_](OH)_2_
Glauconite	K_0.85_ (Fe^3+^,Al^3+^)_1.34_ (Mg^2+^,Fe^2+^)_0.66_☐ [(Al_0.24_Si_3.76_)O_10_](OH)_2_
Montmorillonite	(Na,Ca)_0,3_(Al,Mg)_2_Si_4_O_10_(OH)_2_ • n H_2_O
Gypsum	CaSO_4_,2H_2_O
Calcite	CaCO_3_
Quartz	SiO_4_
Anorthite	Ca(Al_2_Si_2_O_8_)

**Fig. 5:**
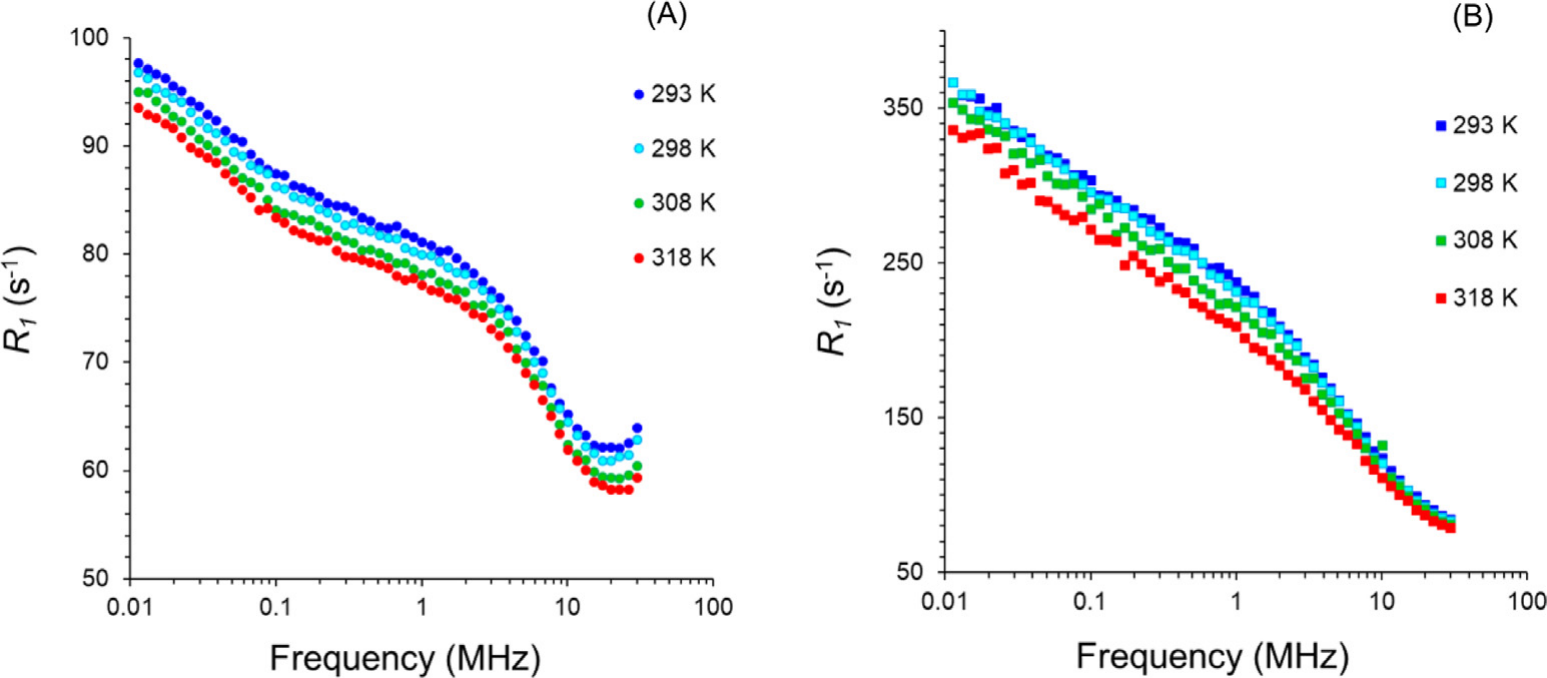
^1^H NMR relaxation profile for Bohemian GE (A) and Cyprus GE (B) at 293k (blue), 298K (cyan), 308K (green) and 318K (red). (For interpretation of the references to color in this figure legend, the reader is referred to the web version of this article.)

The evolution in temperature of the ^1^H NMR relaxation profiles for Bohemian GE and Cyprus GE dispersions (Fig. 5).

**Fig. 6:**
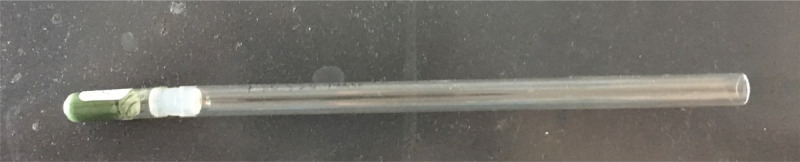
picture of the sample NMR tube with its extension for introduction in the relaxometers.

The sample tube used for conducting the relaxometry experiments is shown in Fig. 6.

## 2 Identification of the main mineral component of France GE

To identify the main component of France GE, X-ray diffraction pattern of France GE powder has been recorded (Fig. 1). The calculated diffraction pattern of gypsum (CaSO_4_,2H_2_O) from the CIF file [2] is also shown.

## 3 Effect of pigment volume fraction

The ^1^H NMR relaxation profiles of water in green earth/water mixtures with different volume fractions are displayed here for several pigments (Fig. 2). The ^1^H relaxation rates measured at frequency spanning from 10 kHz up to 30 MHz were determined by fitting the magnetization evolution versus time using a monoexponential function.

The variation of the relaxation rate *R_1_* versus the volume fraction of pigment *Φ_V_* at 298K has been measured at 20 MHz using the minispec Bruker relaxometer for several pigments.

## 4 Gas adsorption

The specific surface areas were determined using the BET equation whereas the presence of micropores was assessed using the t-plot treatment [3]. Fig. 4 presents the t-plot curves obtained for Bohemian GE and Cyprus GE. It appears that the sample from Bohemian GE displays significantly more micropores then the one from Cyprus GE. This strongly suggests that there are more defects on the edges for the Bohemian GE sample. The values deduced are *S_tot_* = 66 m^2^/g and *S_mic_* = 22 m^2^/g for Bohemian GE and *S_tot_* = 55.7 m^2^/g and *S_mic_* = 11 m^2^/g for Cyprus GE.

## 5 Temperature dependence

The NMR relaxation profile for Boehmian GE and Cyprus GE has been measured for various temperature ranging from 293 to 318K (Fig. 5). For Bohemian GE the whole profile decreases with increasing temperature, while for Cyprus GE, the low frequency part is more affected.

## 6 Experimental design, materials and methods

### 6.1 Samples preparation for NMR measurements

Several mixtures with earth/water ratio ranging between 20 and 60wt% were prepared by mimicking the paint tempera process, i.e. using a glass pigment muller to ensure the homogeneity of the dispersions even in concentrated pasty samples. Powdered green earth was laid on a glass plate, water was spread over it and both components were mixed using the muller until the mixture was homogenous in texture and colour. The mixing time was usually around 2–3 min. In order to prevent evaporation during the experiment, the samples were put in glass tubes 10 mm external in diameter and only 40 mm in length closed by a silicon cap and an extension was built to introduce the tube inside the relaxometer (Fig. 6). Hence, the sample almost completely filled the tube and no evaporation occurs.

### 6.2 X-ray diffraction

The X-ray diffraction pattern of France GE powder has been recorded using a Bruker D8 diffractometer equipped with a copper source emitting with a wavelength of λ_k_1_ = 0.15406 nm and λ_k_2_ = 0.15443 nm. X-ray diffraction patterns were measured using the following parameters: tension of acceleration, 40 kV; current, 40 mA; 2Θ values ranging from 5° to 80°; step, 0.010° and step time, 0.75 s.

### 6.3 NMR relaxometry

The measurements of the water ^1^H longitudinal relaxation rates $R_{1}=\frac{1}{T_{1}}$ have been performed on two different NMR relaxometers. The low frequency domain from 10 kHz to 30 MHz (^1^H frequency) has been explored using a Stelar SpinMaster relaxometer. In this case, *T_1_* has been measured using a pre-polarized sequence from 10 kHz to 10 MHz and a non-polarized sequence from 10 MHz to 30 MHz [4]. The recycle delay was set to 0.5 s, the polarization was done at 20 MHz, the acquisition at 16.35 MHz, the 90° pulse duration was optimized at 9.5 µs. For the *T*_1_ determination, 32 logarithmically spaced recovery delays between approximately 0.01 *T*_1_ and 4 *T*_1_ were used. At 20 MHz, *T_1_* have been measured using a 20 Bruker Minispec using an inversion-recovery sequence with 32 recovery delays ranging from 40 µs to 10 *T_1_* approximately. The 90° pulse duration was optimized at 9.7µs. The samples were thermostated at 298K (except explicitly mentioned in the text) using regulated air flux.

### 6.4 Gas isotherm

Sample gas isotherm are realised with a Micromeritics ASAP 2020 instrument, with 200 mg of sample. Nitrogen adsorption isotherms were carried out at 77 K after outgassing the samples at 323 K for 24 h.

## Declaration of Competing Interest

The authors declare that they have no known competing financial interests or personal relationships which have, or could be perceived to have, influenced the work reported in this article.
